# Dataset of mitochondrial genome variants associated with asymptomatic atherosclerosis

**DOI:** 10.1016/j.dib.2016.04.055

**Published:** 2016-04-29

**Authors:** Margarita A. Sazonova, Andrey V. Zhelankin, Valeria A. Barinova, Vasily V. Sinyov, Zukhra B. Khasanova, Anton Y. Postnov, Igor A. Sobenin, Yuri V. Bobryshev, Alexander N. Orekhov

**Affiliations:** a Institute of General Pathology and Pathophysiology Russian Academy of Medical Sciences, Moscow, Russian Federation; b Laboratory of Medical Genetics, Russian Cardiology Research and Production Complex, Moscow, Russian Federation; c Faculty of Medicine, School of Medical Sciences, University of New South Wales, Sydney, Australia; d Institute for Atherosclerosis Research, Skolkovo Innovative Centre, Moscow Region, Russian Federation; e Department of Biophysics, Biological Faculty, Moscow State University, Moscow, Russian Federation

**Keywords:** mtDNA variant, Mitochondrial genome, Homoplasmic, Asymptomatic atherosclerosis

## Abstract

This dataset report is dedicated to mitochondrial genome variants associated with asymptomatic atherosclerosis. These data were obtained using the method of next generation pyrosequencing (NGPS). The whole mitochondrial genome of the sample of patients from the Moscow region was analyzed. In this article the dataset including anthropometric, biochemical and clinical parameters along with detected mtDNA variants in patients with carotid atherosclerosis and healthy individuals was presented. Among 58 of the most common homoplasmic mtDNA variants found in the observed sample, 7 variants occurred more often in patients with atherosclerosis and 16 variants occurred more often in healthy individuals.

**Specifications table**

**Table:** 

Subject area	*Genetics, Molecular genetics*
More specific subject area	*Atherosclerosis*
Type of data	*Sequencing data*
How data was acquired	*Roche 454 GS Junior Titanium*
Data format	*Raw, analysed*
Experimental factors	*Ultrasound scanning of carotid arteries; extraction of DNA from blood leucocytes of patients; enrichment of mitochondrial genome by the amplification of whole mitochondrial genome; sequencing of mtDNA*
Experimental features	*We performed next generation pyrosequencing of whole mitochondrial genome of the sample of patients from the Moscow region with carotid atherosclerosis and control subjects without atherosclerosis. mtDNA variants were characterized by the occurrence in the compared groups.*
Data source location	*Moscow, Russian Federation*
Data accessibility	*Data is with this article.*

**Value of the data**

• The data of next generation pyrosequencing of whole mitochondrial genome let us find homoplasmic variants associated with atherosclerotic lesions of carotid arteries in patients from the Moscow region with atherosclerosis and subjects without atherosclerotic lesions of carotid arteries.
• mtDNA sequencing data was obtained using Roche 454 GS Junior Titanium system.
• The data of statistical analysis was obtained using IBM SPSS Statistics v.21.0.
• The data helps to detect new genetic markers of individual predisposition to asymptomatic atherosclerosis in humans.

## 1 Data

**Fig. 1:**
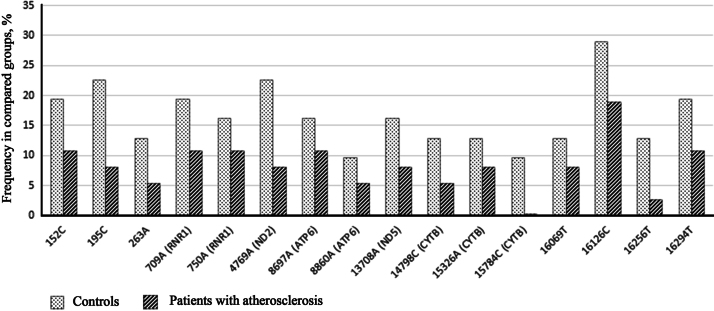
The most common mtDNA variants that were more frequent (with ratio >=1.5) in controls compared with atherosclerotic patients. For positions in coding region, names of genes are given in brackets.

**Fig. 2:**
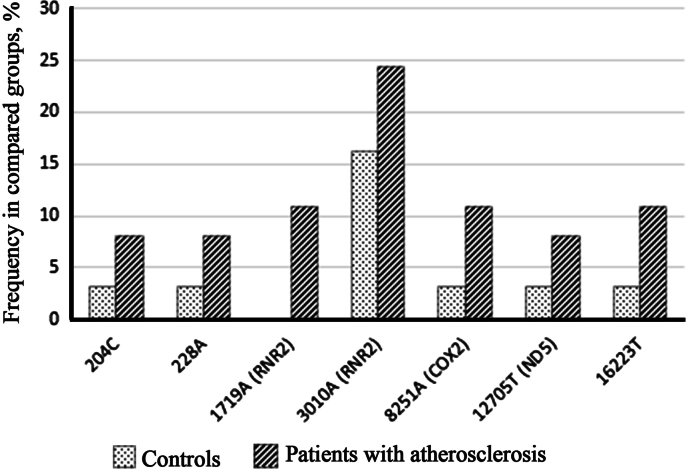
The most common mtDNA variants that were more frequent (with ratio >=1.5) in atherosclerotic patients compared with controls. For positions in coding region, names of genes are given in brackets.

The relevant data is provided in this article. See Table 1 and Table 1, and Fig. 1, Fig. 2. The raw data files (fastq files) that were used in the analysis and interpretation are available in Institute for Atherosclerosis Research, Skolkovo Innovative Center, Moscow Region, Russian Federation. http://inat.ru/.

## 2 Experimental design, materials and methods

This dataset report is dedicated to mtDNA variants, associated with asymptomatic atherosclerosis. These data were obtained using the method of next generation pyrosequencing (NGPS). The whole mitochondrial genome of the sample of patients from the Moscow region was analyzed.

In this article the dataset of homoplasmic mtDNA variants in patients with atherosclerosis and healthy individuals from the Moscow region was presented (Table 1 and Table 1, Fig. 1, Fig. 2).

### 2.1 Materials

The materials for obtaining the data were leukocytes from whole blood of 68 patients. 31 subjects with carotid atherosclerosis and 37 control subjects without atherosclerosis were selected for the study [1], [2], [3], [4], [5] (Table 1).

Selected individuals did not have severe clinical manifestations of atherosclerosis and oncological diseases. The number of patients with diabetes mellitus was minimized (3 subjects among the sample).

### 2.2 Methods

To assess the state of the carotid artery wall, high-resolution B-mode ultrasonography was performed with ultrasound scanner SonoScape SSI-1000 (China) using a linear vascular 7.5 MHz sensor. Values of the average carotid intima-medial thickness (CIMT) were used to estimate the presence and severity of atherosclerotic plaques in carotid arteries (Table 1). Borderline age-related CIMT values for Moscow region population were used to characterize the presence of carotid atherosclerosis [6]. If there was a presence of atherosclerotic plaque with the carotid artery stenosis of more than 20% or thickening of the intima-media layer exceeding the boundaries of the 75th percentile, as well as the combination of these factors, persons were considered as belonging to the group of patients with atherosclerosis. Controls were characterized by CIMT values that does not exceed median values for appropriate age group, and by the absence of atherosclerotic plaques (for some persons – the presence of carotid stenosis that does not exceed 20%).

The extraction of DNA from blood leukocytes of patients was performed using methods developed earlier by us [7], [8], [9], on the basis of the methods, published by Maniatis et al. [10], [11]. Before sequencing, an enrichment of mitochondrial genome by the amplification of the whole mitochondrial genome using REPLI-g Mitochondrial Kit (Qiagen, Germany) was performed. To carry out mtDNA sequencing, Roche 454 GS Junior Titanium system (Roche Applied Science, USA) was used. Sequencing workflow was performed according to the manufacturer׳s recommendations and using appropriate instruments and reagents.

Sequence analysis of mitochondrial DNA was carried out using GS Reference Mapper software (Roche Applied Science, USA). Cambridge reference sequence of the human mitochondrial genome (rCRS, NC_012920.1) was used for mapping [12]. Statistical analysis of the obtained data was carried out using IBM SPSS Statistics v.21.0.

### 2.3 Data obtained with the use of NGPS

We identified 58 most common homoplasmic variants that were characterized by more than 5% presence in the observed sample (Table 1). Among them, 7 mtDNA variants were associated with presence of atherosclerotic lesions of carotid arteries (204C, 228A, 1719A, 3010A, 8251A, 12705T, 16223T) (Fig. 1) and 16 variants mtDNA occurred more often in healthy individuals (152T, 195C, 263A, 709A, 750A, 4769A, 8697A, 8860A, 13708A, 14798C, 15326A, 15784С, 16069T, 16126C, 16256T, 16294T) (Fig. 2).

## Figures and Tables

**Table 1:** The most common homoplasmic mtDNA variants in the observed sample.

mtDNA variant	Gene/region	Character of mutation^a^	% of cases		
General	Controls	Atherosclerotic patients
m.73A>G	Noncoding	–	51.5	51.6	51.4
m.146T>C	Noncoding	–	7.4	6.5	8.1
m.152T>C	Noncoding	–	14.7	19.4	10.8
m.185G>A	Noncoding	–	5.9	6.5	5.4
m.195T>C	Noncoding	–	14.7	22.6	8.1
m.204T>C	Noncoding	–	5.9	3.2	8.1
m.228G>A	Noncoding	–	5.9	3.2	8.1
m.263A>G	Noncoding	–	91.2	87.1	94.6
m.489T>C	Noncoding	–	7.4	6.5	8.1
m.709G>A	RNR1	–	14.7	19.4	10.8
m.750A>G	RNR1	–	86.8	83.9	89.2
m.930G>A	RNR1	–	7.4	6.5	8.1
m.1438A>G	RNR1	–	83.8	83.9	83.8
m.1719G>A	RNR2	–	5.9	0.0	10.8
m.1811A>G	RNR2	–	13.2	12.9	13.5
m.1888G>A	RNR2	–	11.8	12.9	10.8
m.2706A>G	RNR2	–	50.0	45.2	54.1
m.3010G>A	RNR2	–	20.6	16.1	24.3
m.3107delN	RNR2	–	89.7	83.9	94.6
m.4216T>C	ND1	m	20.6	22.6	18.9
m.4769A>G	ND2	s	85.3	77.4	91.9
m.4917A>G	ND2	m	14.7	16.1	13.5
m.5147G>A	ND2	s	8.8	9.7	8.1
m.7028C>T	COX1	s	48.5	48.4	48.6
m.8251G>A	COX2	s	7.4	3.2	10.8
m.8697G>A	ATP6	s	13.2	16.1	10.8
m.8860A>G	ATP6	m	92.6	90.3	94.6
m.10398A>G	ND3	m	16.2	19.4	13.5
m.10463T>C	TRNR	–	14.7	16.1	13.5
m.11251A>G	ND4	s	19.1	22.6	16.2
m.11467A>G	ND4	s	19.1	19.4	18.9
m.11719G>A	ND4	s	50.0	51.6	48.6
m.11812A>G	ND4	s	10.3	9.7	10.8
m.12308A>G	TRNL2	–	20.6	22.6	18.9
m.12372G>A	ND5	s	20.6	22.6	18.9
m.12612A>G	ND5	s	8.8	9.7	8.1
m.12705C>T	ND5	s	5.9	3.2	8.1
m.13368G>A	ND5	s	14.7	16.1	13.5
m.13708G>A	ND5	m	11.8	16.1	8.1
m.14233A>G	ND6	s	11.8	9.7	13.5
m.14766C>T	CYTB	m	45.6	48.4	43.2
m.14798T>C	CYTB	m	8.8	12.9	5.4
m.14905G>A	CYTB	s	14.7	16.1	13.5
m.15326A>G	CYTB	m	89.7	87.1	91.9
m.15452C>A	CYTB	m	22.1	25.8	18.9
m.15607A>G	CYTB	s	11.8	12.9	10.8
m.15784T>C	CYTB	s	4.4	9.7	0.0
m.15928G>A	TRNT	–	11.8	12.9	10.8
m.16069C>T	Noncoding	–	10.3	12.9	8.1
m.16126T>C	Noncoding	–	23.5	29.0	18.9
m.16145G>A	Noncoding	–	5.9	6.5	5.4
m.16223C>T	Noncoding	–	7.4	3.2	10.8
m.16256C>T	Noncoding	–	7.4	12.9	2.7
m.16294C>T	Noncoding	–	14.7	19.4	10.8
m.16296C>T	Noncoding	–	8.8	9.7	8.1
m.16304T>C	Noncoding	–	11.8	9.7	13.5
m.16311T>C	Noncoding	–	10.3	6.5	13.5
m.16519T>C	Noncoding	–	51.5	51.6	51.4

a For mutations in protein coding genes: m – missense, s – synonymous.

## Appendix A Supplementary material

Supplementary material

Supplementary material **Table 1**. Available data about subjects used in the study.
