# Supplementary material for: Dataset of mitochondrial genome variants associated with asymptomatic atherosclerosis
Source: Data Brief. 2016 Apr 29;7:1570–5. doi: 10.1016/j.dib.2016.04.055 (PMC4865666; doi:10.1016/j.dib.2016.04.055)
Supplement: Supplementary file 1 — Supplementary material [file mmc1.doc]

**Conflict of interest disclosure**

The authors declare that the research was conducted in the absence of any commercial or financial relationships that could be construed as a potential conflict of interest.
